# Peripancreatic Tuberculosis Mimicking Metastatic Pancreatic Malignancy With Hepatic Lesions

**DOI:** 10.7759/cureus.111776

**Published:** 2026-06-29

**Authors:** Sorour Raeiskarimi, Gordafarin Nikbakht, Fariba Zarei

**Affiliations:** 1 Shiraz Medical School, Shiraz University of Medical Sciences, Shiraz, IRN; 2 Department of Infectious Diseases, Yasuj University of Medical Sciences, Yasuj, IRN; 3 Department of Radiology, Medical Imaging Research Center, Shiraz University of Medical Sciences, Shiraz, IRN

**Keywords:** abdominal tuberculosis, acid-fast bacilli, caseating granuloma, extrapulmonary tuberculosis, hepatic lesions, pancreatic malignancy, pancreatic mass, peripancreatic tuberculosis, polymerase chain reaction

## Abstract

A 61-year-old woman presented with chronic constipation, epigastric pain, intermittent fever, night sweats, anorexia, and marked weight loss. Imaging showed porta hepatis and peripancreatic lymphadenopathy, small hepatic lesions, and a pancreatic-region lesion, raising concern for metastatic pancreatic malignancy or lymphoma. The initial liver biopsy was negative for metastatic carcinoma and lymphoma. Surgery was considered but canceled after magnetic resonance cholangiopancreatography did not confirm a definite pancreatic mass requiring operative management. A repeat core biopsy of the presumed pancreatic-region lesion sampled lymph node tissue and showed caseating granulomatous inflammation with positive acid-fast staining. PCR testing later confirmed *Mycobacterium tuberculosis* complex DNA. Four-drug antituberculous therapy was started before PCR confirmation because the pathology and acid-fast staining strongly supported tuberculosis. The patient improved clinically and biochemically after treatment. This case supports that repeat targeted tissue sampling should be pursued when malignancy-like pancreatic-region findings are discordant with pathology, particularly in tuberculosis-endemic settings.

## Introduction

Peripancreatic tuberculosis is an uncommon form of extrapulmonary tuberculosis that may appear as a pancreatic-region mass with regional lymphadenopathy. Abdominal tuberculosis more often involves the gastrointestinal tract, peritoneum, lymph nodes, or solid abdominal organs, while pancreatic or peripancreatic involvement is unusual [1,2]. Symptoms are usually non-specific and may include abdominal pain, fever, night sweats, anorexia, weight loss, jaundice, or vomiting [1-3].

The main clinical problem is its resemblance to pancreatic malignancy. Imaging may show a solid or cystic pancreatic lesion, peripancreatic or porta hepatis lymphadenopathy, biliary obstruction, or lesions in adjacent organs [2-4]. These findings can lead to a presumptive diagnosis of pancreatic cancer, lymphoma, or metastatic disease, especially in older adults. Tissue diagnosis is central when imaging findings and the clinical course do not fully match malignancy. Endoscopic ultrasound-guided fine-needle aspiration or biopsy is often used when a pancreatic or peripancreatic lesion is accessible, while a CT-guided or ultrasound-guided biopsy may be appropriate depending on lesion location and local expertise [1,5,6].

We report a case of biopsy-confirmed peripancreatic tuberculosis in a 61-year-old woman who presented with a suspected pancreatic mass, porta hepatis lymphadenopathy, and multiple hepatic lesions initially interpreted as possible metastatic disease. The diagnosis was established after a repeat core biopsy showed caseating granulomatous lymphadenitis with positive acid-fast staining, followed by confirmation of *Mycobacterium tuberculosis* complex DNA using polymerase chain reaction.

## Case presentation

A 61-year-old postmenopausal woman from a rural area of southwestern Iran was evaluated for chronic constipation, epigastric abdominal pain, intermittent fever, chills, night sweats, anorexia, and weight loss. She had no known history of diabetes mellitus, malignancy, immunosuppressive disease, or previous tuberculosis. She reported no known household or occupational contact with a patient with tuberculosis. She had chronic low back pain and intermittent weekly hookah use. She denied alcohol or opium use. She also reported a daily cough but had not previously been diagnosed with pulmonary tuberculosis.

The patient had longstanding constipation and underwent a colonoscopy in October 2024, which was normal. Approximately one month later, she gradually developed epigastric pain, fever, chills, and night sweats. These symptoms persisted and worsened over the following months. She was admitted to a local hospital in Yasuj for approximately one week, where a tuberculin skin test was reportedly negative. No diagnosis of tuberculosis or malignancy was established during that admission.

The patient lived in a rural area with limited local access to advanced diagnostic procedures. Financial barriers and sequential referrals between Yasuj, Isfahan, and Shiraz contributed to the length of the diagnostic workup. During this period, her oral intake declined. She later reported marked anxiety after being told that malignancy was possible, with poor oral intake, postprandial nausea and vomiting, and progressive weight loss from 89 kg to 70 kg. She also reported mild scleral icterus and mild pruritus. She did not report dark urine or pale stools. Abdominal examination during her evaluations showed tenderness without a palpable mass. No peripheral lymphadenopathy was found.

Initial abdominopelvic ultrasonography in January 2025 showed grade I hepatic steatosis and several prominent lymph nodes in the pancreaticoduodenal region and porta hepatis, with a maximum short-axis diameter of 17.5 mm. Contrast-enhanced abdominopelvic computed tomography (CT) performed in February 2025 demonstrated several prominent lymph nodes in the pancreaticoduodenal, portacaval, and porta hepatis regions, with maximum short-axis diameters of 14.5, 11.5, and 9 mm, respectively. The liver, pancreas, biliary system, gallbladder, kidneys, bowel loops, and pelvic organs were otherwise reported as normal.

A subsequent abdominal and pelvic CT with dynamic liver protocol in February 2025 showed multiple small hypoattenuating lesions in both hepatic lobes, the largest measuring 8 mm. These lesions were interpreted as highly suspicious for secondary metastases or lymphoma. Multiple porta hepatis lymph nodes were also identified, measuring up to 22 × 15 mm (Figure 1). Limited evaluation of the lower chest showed subcarinal and right hilar lymphadenopathy.

**Figure 1:**
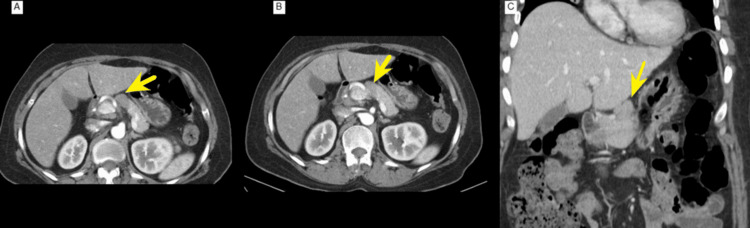
Dynamic contrast-enhanced abdominopelvic CT images showing porta hepatis and peripancreatic lymphadenopathy, indicated by arrowheads.

Because the hepatic lesions were interpreted as possible metastatic targets, a hepatic micronodule was selected for core needle biopsy in March 2025. Histopathologic examination showed normal liver parenchyma and was negative for metastatic carcinoma, lymphoma, and hepatic steatosis. This result reduced the likelihood of metastatic disease but did not explain the persistent porta hepatis and peripancreatic lymphadenopathy. Sampling error remained possible because the hepatic lesions were small, with the largest measuring 8 mm.

Follow-up ultrasonography in June 2025 again showed a pancreatic-region abnormality, interpreted as either a pancreatic mass or adjacent lymphadenopathy. Magnetic resonance cholangiopancreatography (MRCP) performed later in 2025 again showed numerous hepatic lesions suspicious for secondary malignancy and enlarged lymph nodes in the porta hepatis and along the common hepatic artery, measuring up to 21 × 24 mm. No definite pancreatic mass was visualized, and the common hepatic duct, common bile duct, gallbladder, and ampullary region were unremarkable. Endoscopic ultrasound was suggested if clinically indicated. Surgery had been planned because of concern for pancreatic malignancy. The operation was canceled after MRCP did not confirm a clear pancreatic mass or biliary obstruction requiring operative management. The patient was discharged without a definitive diagnosis, and further evaluation continued because symptoms and imaging abnormalities persisted.

In December 2025, abdominal and transvaginal ultrasonography showed a heterogeneous, coarse liver echopattern and a 17 × 15 mm solid hypoechoic lesion in segment VII of the right hepatic lobe. A vascularized solid hypoechoic lesion measuring approximately 40 × 20 mm was identified in the pancreatic body, and triphasic CT was recommended. Laboratory testing around this time showed leukocytosis with a white blood cell count of 14,400/µL, thrombocytosis with a platelet count of 490,000/µL, and anemia with hemoglobin of 10.4 g/dL. Alkaline phosphatase was markedly elevated at 746 U/L, while aspartate aminotransferase and alanine aminotransferase were 28 U/L and 22 U/L, respectively. Serum ferritin was 700.05 ng/mL. Tumor markers, including carcinoembryonic antigen and carbohydrate antigen 19-9, were negative. Human immunodeficiency virus testing was also negative.

A repeat core biopsy of the presumed pancreatic-region mass was performed in January 2026. The specimen consisted of three cores of soft tissue, measuring 1.8 × 0.1 × 0.1 cm in aggregate. Histopathology showed lymph node tissue with caseating granulomatous inflammation. Acid-fast staining was positive. Because the sampled tissue was lymph node tissue rather than pancreatic parenchyma, the diagnosis was considered peripancreatic tuberculous lymphadenitis presenting radiologically as a pancreatic-region mass.

The patient reported a daily cough during the illness. No sputum acid-fast bacilli result was available in the reviewed records before the tissue diagnosis. The initial workup was directed toward suspected abdominal malignancy because the dominant findings were hepatic lesions, porta hepatis lymphadenopathy, and a pancreatic-region lesion. After the biopsy supported tuberculosis, she was managed by the infectious disease team according to local tuberculosis practices.

The infectious disease team started rifampin, isoniazid, pyrazinamide, and ethambutol with pyridoxine 40 mg daily before PCR confirmation. Treatment was started at the first infectious disease visit because the pathology showed caseating granulomatous lymphadenitis, acid-fast staining was positive, tuberculosis was clinically plausible in the local setting, and PCR testing was not immediately feasible. Qualitative real-time PCR performed on DNA extracted from the paraffin-embedded biopsy block later detected *Mycobacterium tuberculosis* complex DNA, confirming the diagnosis.

The patient tolerated treatment without major adverse effects. She reported improved appetite, resolution of fever and chills, and overall clinical recovery after starting therapy. She noted orange discoloration of the urine, consistent with rifampin use, and intermittent paresthesia in the extremities. Follow-up laboratory testing in April 2026 showed improvement in inflammatory and hematologic markers, with a white blood cell count of 6,700/µL, platelet count of 250,000/µL, hemoglobin of 11.8 g/dL, erythrocyte sedimentation rate of 15 mm/hour, and negative C-reactive protein. Alkaline phosphatase decreased to 264 U/L, and bilirubin levels were normal. Follow-up abdominal ultrasonography in April 2026 showed mild grade I fatty liver and a residual hypoechoic lesion measuring 20 × 25 mm at the pancreatic neck. No biliary dilatation, ascites, hydronephrosis, or abdominal free fluid was reported.

## Discussion

Peripancreatic tuberculosis is uncommon, even in settings where tuberculosis is prevalent. The pancreas is an unusual site of involvement compared with the gastrointestinal tract, peritoneum, intra-abdominal lymph nodes, liver, and spleen [1,2]. Clinical manifestations are often non-specific and may include abdominal pain, fever, night sweats, anorexia, weight loss, vomiting, jaundice, or a pancreatic-region mass on imaging [1-4]. Our patient presented with epigastric pain, fever, night sweats, marked weight loss, mild scleral icterus, porta hepatis lymphadenopathy, and a lesion repeatedly interpreted as a pancreatic or peripancreatic mass, appropriately raising concern for pancreatic malignancy.

The diagnostic challenge in this case arose from discordant imaging and pathology. CT, MRCP, and ultrasonography repeatedly demonstrated hepatic lesions and a pancreatic-region abnormality suspicious for metastatic disease or lymphoma, although similar findings may also occur in extrapulmonary tuberculosis [7-9]. The initial core biopsy of a hepatic micronodule showed normal liver parenchyma and did not confirm malignancy. However, because the targeted lesions were small, sampling error remained possible, and the negative result did not explain the persistent porta hepatis and peripancreatic lymphadenopathy or the enlarging pancreatic-region lesion. Continued constitutional symptoms, negative tumor markers, and residence in a tuberculosis-endemic region supported further tissue sampling before surgery. A repeat biopsy of the presumed pancreatic-region lesion ultimately sampled lymph node tissue and demonstrated caseating granulomatous inflammation with positive acid-fast staining, establishing peripancreatic tuberculous lymphadenitis presenting as a pancreatic-region mass.

Several clinical features further supported consideration of tuberculosis. The patient had constitutional symptoms, abdominal lymphadenopathy, negative tumor markers, negative human immunodeficiency virus testing, and residence in an area where tuberculosis remains clinically relevant. A negative tuberculin skin test did not exclude tuberculosis. Although these findings were not diagnostic, they reinforced the need for repeat tissue sampling rather than proceeding directly to major surgery. Previous reports have similarly emphasized that pancreatic and peripancreatic tuberculosis may only be diagnosed after biopsy, aspiration, or surgery when malignancy is initially suspected [3-6,10].

Tissue diagnosis remains central in this setting. Endoscopic ultrasound-guided fine-needle aspiration or biopsy is often used when a pancreatic or peripancreatic lesion is accessible, whereas CT-guided or ultrasound-guided sampling may be appropriate depending on lesion location and available expertise [1,5,6,10]. Histopathology may demonstrate granulomatous inflammation with or without caseation, and acid-fast bacilli are not always identified; therefore, culture or nucleic acid amplification testing can improve diagnostic confidence when available [5,6]. In our patient, caseating granulomatous lymphadenitis, positive acid-fast staining, and PCR detection of *Mycobacterium* *tuberculosis* complex DNA established the diagnosis. Starting antituberculous therapy before PCR confirmation was clinically justified because pathology and acid-fast staining strongly supported tuberculosis and molecular testing was not immediately available.

The patient’s clinical and laboratory response further supported the diagnosis. After initiation of rifampin, isoniazid, pyrazinamide, and ethambutol with pyridoxine, she experienced resolution of fever and chills, improved appetite, normalization of leukocytosis and thrombocytosis, an increase in hemoglobin concentration, and a marked decline in alkaline phosphatase. A residual hypoechoic lesion remained on early follow-up ultrasonography, a finding that may precede complete radiologic resolution.

This case highlights an important practical lesson. A pancreatic-region lesion accompanied by hepatic abnormalities and regional lymphadenopathy should not automatically be assumed to represent advanced malignancy, particularly in tuberculosis-endemic settings. When imaging findings are discordant with pathology or when an initial biopsy is non-diagnostic, repeat sampling of the most informative persistent lesion may prevent unnecessary surgery and facilitate earlier diagnosis and appropriate antituberculous treatment.

## Conclusions

Peripancreatic tuberculosis can closely resemble metastatic pancreatic malignancy when imaging shows a pancreatic-region mass, regional lymphadenopathy, and hepatic lesions. In this patient, the radiologic findings led to concern for advanced malignancy, while the initial liver biopsy did not provide an explanation for the persistent lymphadenopathy or pancreatic-region lesion. Repeat biopsy was necessary to establish the correct diagnosis.

Tuberculosis should remain in the differential diagnosis of pancreatic or peripancreatic masses in patients with constitutional symptoms, lymphadenopathy, negative tumor markers, or residence in an endemic region. When imaging and pathology do not match, repeat tissue sampling from the most informative lesion can prevent avoidable surgery. Histopathology, acid-fast staining, and molecular testing ultimately established the diagnosis and allowed appropriate antituberculous treatment. The prolonged diagnostic interval in this case supports earlier repeat targeted sampling when pancreatic-region imaging suggests malignancy, but initial pathology is non-confirmatory.
